# Supplementary material for: Hierarchical approach to evaluating storage requirements for renewable-energy-driven grids
Source: iScience. 2022 Dec 28;26(1):105900. doi: 10.1016/j.isci.2022.105900 (PMC9852348; doi:10.1016/j.isci.2022.105900)
Supplement: Document S1. Figure S1 and Table S1 [file mmc1.pdf]

iScience, Volume *26*

Supplemental information

Hierarchical approach to evaluating storage requirements for renewable-energy-driven grids

Zabir Mahmud, Kenji Shiraishi, Mahmoud Y. Abido, Pedro Andrés Sánchez-Pérez, and Sarah R. Kurtz

Supplementary Material for "Hierarchical Approach to Evaluating Storage Requirements for Renewable-Energy-Driven Grids"

Zabir Mahmud[1], Kenji Shiraishi[2], Mahmoud Y. Abido[1, 3], P. A. Sánchez-Pérez[1], and Sarah Kurtz[1]

[1]University of California, Merced, 5200 N Lake Rd, Merced, CA 95343, United States
[2]University of California, Berkeley, 450 Sutardja Dai Hall, Berkeley, CA 94720, United States
[3]Cairo University, Giza, 1 Gamaa Street, Giza, Cairo 12613, Egypt

1 Energy Resources and Description of Modeled Sites

We explain how we consider sites for different scenarios. In Table S1, we mention the list of specific latitude and longitude with the region names modelled in the analysis.

Table S1: Description of the renewable electricity generators with their names, locations and modeling parameters. Related to STAR Method

	Modeled sites			
Energy resource	Name	Latitude	Longitude	Modeling assumptions
**Solar PV**				
Historical (2019)	CAISO data [1]	-	-	Module type: Standard,
Latitude tilt	Los Angeles	34.58	-118.16	Array type: 1-Axis tracking,
	Tehachapi	35.15	-118.44	Total system losses: 14.08%,
	Merced	37.29	-120.52	DC to AC ratio: 1.2,
				Data collected: 2012,
Zero tilt	Same locations as latitude tilt			Data type: hourly
**Onshore wind**				
Historical (2019)	CAISO data [1]	-	-	Turbine power curve: GE 2.5-120,
Summer dominant	Ocotillo	32.87	-115.55	Hub height: 100m,
	San Gorgonio	33.88	-116.56	Data collected: 2012,
	Alta I	35.02	-118.25	Data type: 5-min interval
	Shiloh I	38.12	-121.79	
Winter dominant	Golden Acorn Casino	32.70	-116.35	
	Tehachapi	35.14	-118.45	
	Carrizo	34.99	-120.15	
	Foundation Walmart	40.01	-122.13	
	Humboldt	40.76	-123.87	
**Offshore wind**				
Northern	Cape Mendocino	40.43	-124.41	Turbine power curve:
	Humboldt Bay	40.90	-124.37	Aerodyn SCD 8.0/168,
	Del Norte	41.63	-124.23	Hub height: 120m,
Southern	Diablo Canyon	35.13	-120.99	Data collected: 2019,
	Morro Bay	35.33	-121.01	Data type: 5-min interval

2 Sensitivity Analysis of Total Generation Amount

The generation is considered 120% of total annual load in the main paper. However, the total generation could be different in future. We analyze the impact of different generation level on energy storage statistics as shown in Figure S1. We see that the seasonal needs could be reduced by 4 times if the generation increases from 120% to 180% of total annual load though the effect on the required diurnal storage is relatively small.

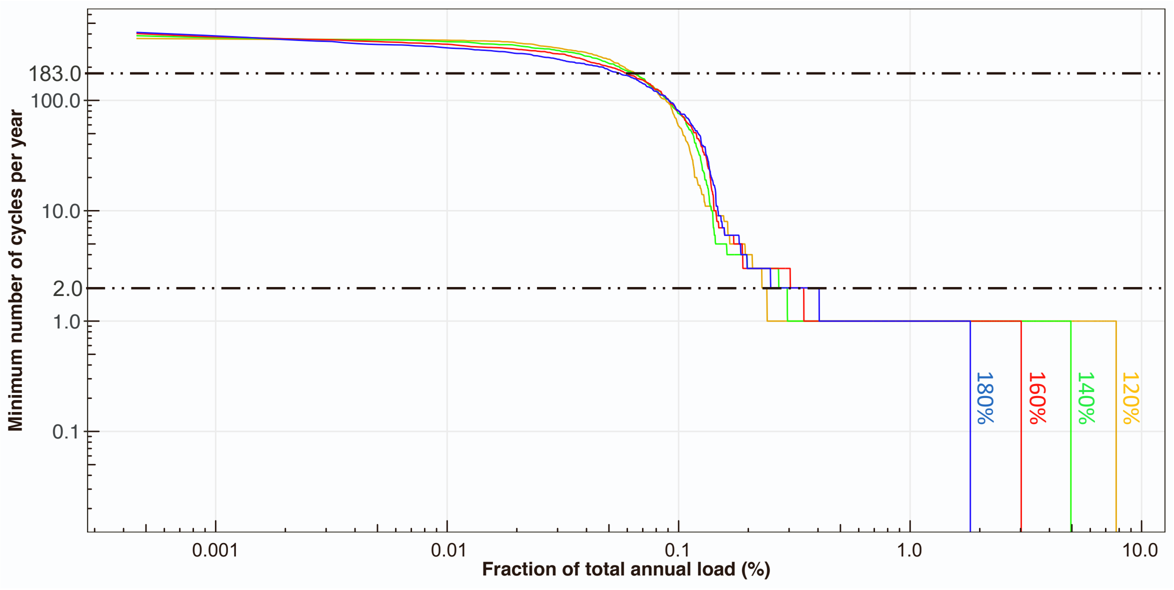

Figure S1: Sensitivity analysis of amount of total generation for baseline scenario energy mix. Related to Figure 1

3 General Equations

We calculate the eight generation profiles using Equations 1 and 2. Renewables include solar, wind, biomass, biogas, geothermal and small hydropower in Equation 2. Initially charge is calculated for each time step, t, by the difference between total generation and load as shown in Equation 3. Considering the charging and discharging efficiency, charges are recalculated using Equations 4 and 5. The state of charge of each storage bin is determined using the cumulative summation of charge after considering the dis/charging for 105,120 timesteps as shown in Equation 6.

$$\text{Total generation} = \text{Historical (2019) generation} + \text{Added generation} \tag{1}$$

$$\text{Historical (2019) generation} = \text{Renewables} + \text{Large hydro} \tag{2}$$

$$\text{Available Charge (t)} = \text{Total generation (t)} + \text{Load (t)} \; t = \{1, 2, \ldots, 105120\} \tag{3}$$

$$\text{Charge (t)} = \text{Available charge (t)} \times \text{Charging efficiency} \tag{4}$$

$$\text{Charge (t)} = \text{Available charge (t)} \div \text{Discharging efficiency} \tag{5}$$

$$\text{State of charge (bin,t)} = \text{State of charge (bin,t-1)} + \text{Charge (t)} \tag{6}$$
