# Supplementary material for: Hierarchical approach to evaluating storage requirements for renewable-energy-driven grids
Source: iScience. 2022 Dec 28;26(1):105900. doi: 10.1016/j.isci.2022.105900 (PMC9852348; doi:10.1016/j.isci.2022.105900)
Supplement: Document S2. Article plus supplemental information [file mmc2.pdf]

iScience

CellPress
OPEN ACCESS

Article

Hierarchical approach to evaluating storage requirements for renewable-energy-driven grids

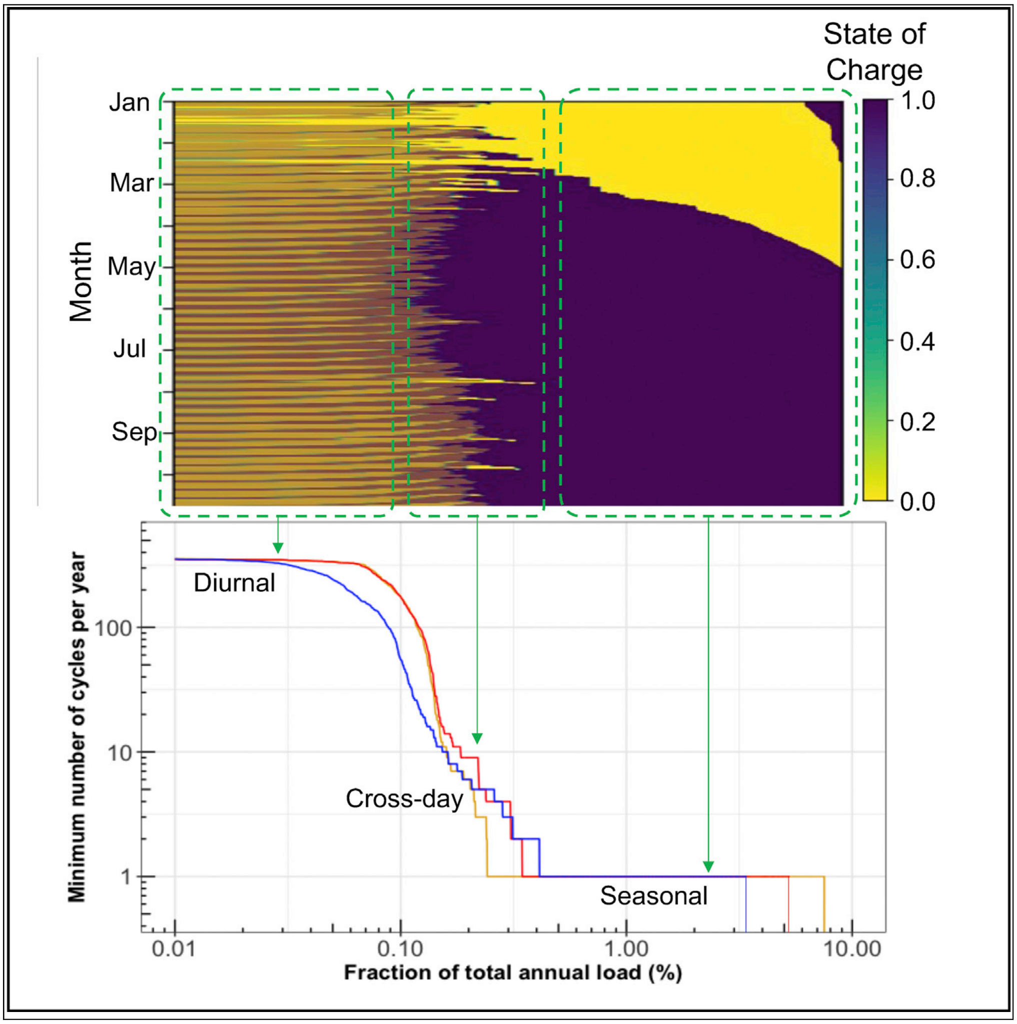

Zabir Mahmud, Kenji Shiraishi, Mahmoud Y. Abido, Pedro Andrés Sánchez-Pérez, Sarah R. Kurtz

zabirsami@gmail.com

Highlights

Hierarchical method estimates the minimum need for amount and frequency of storage use

Storage needs for a renewables-driven grid depend on the generation profiles

Adding more wind in the grid energy mix decreases diurnal storage use

Using onshore wind that is more prevalent in winter can halve seasonal storage needs

Mahmud et al., iScience *26*, 105900
January 20, 2023 © 2022 The Authors.
https://doi.org/10.1016/j.isci.2022.105900

Check for updates

iScience

CellPress
OPEN ACCESS

Article

Hierarchical approach to evaluating storage requirements for renewable-energy-driven grids

Zabir Mahmud,[1,5,*] Kenji Shiraishi,[2] Mahmoud Y. Abido,[3,4] Pedro Andrés Sánchez-Pérez,[1] and Sarah R. Kurtz[1]

SUMMARY

Energy storage can accelerate the decarbonization of the electrical grid. As useful energy storage technologies are developed, investors and manufacturers want to determine the needs for storage in a wide range of scenarios. In this study, we introduce a strategy for identifying the types of storage that will be most valuable to the grid given specific generation and load profiles. This method estimates the annual minimum number of cycles for each storage, how long each holds the charge, and charging and discharging rates for an idealized system, giving insight into tomorrow's complex systems. We demonstrate the proposed hierarchical approach and quantify how many fewer times wind-driven grids cycle the storage at night compared with solar-driven grids, as well as how winter-dominant wind generation and latitude-tilt solar may reduce the need for seasonal storage. Also, we quantify how higher discharging rates are required for energy storage products that cycle most frequently.

INTRODUCTION

One of the most significant steps to slow carbon dioxide emissions is to replace fossil fuels with renewable resources for electricity generation, stimulating greater interest in energy storage. Opportunities for energy storage technologies are even broader, including storage to help nuclear power plants balance supply with variable electricity demand and storage to reduce congestion (e.g., lower demand charges and less need to build out transmission). Many countries are adopting solar, wind, hydro, geothermal, and other renewable energy resources to achieve a low-carbon future[1,2] as anticipated to be an attractive and sustainable approach to limiting the effects of climate change.[3] Many storage technologies are being developed,[4–6] but the optimal storage configurations will depend on which of many possible generations and load profiles turn out to be relevant.

Pumped-hydro storage has historically dominated utility-scale electricity storage with relatively high efficiencies (about 80% round trip) and excellent lifetime.[7] Some researchers have proposed that pumped-hydro storage systems, especially off-river designs,[8,9] could meet the need for storage. Still, there is little evidence that this solution will be implemented fast enough. Li-ion battery prices are dropping quickly,[10] and utilities are now installing them at the GW scale,[11] but they have limited lifetimes,[12] and it's not yet clear whether Li-ion battery prices will be able to fall low enough and be scaled to adequate size to be used for all applications. Promising storage technologies have been described in the literature with tabulations of performance (efficiency, response time, idle losses, lifetime) and cost related to both the power and energy rating.[5,7] As funding agencies invest in technology development and companies develop specific products, questions arise about the optimal design of the storage, especially concerning the ratio of energy rating to the power rating, which we refer to here as the "duration". While it is clear that higher efficiency, lower cost, and longer lifetime are beneficial to any technology, it is less clear whether there is an optimal duration (number of hours a storage element may operate at rated power before running out of stored energy) that funding agencies should prioritize for technology development or that companies should prioritize for commercialization. As shown by multiple studies,[10,13–15] different grid roles need different types of storage attributes, like duration. It is probable that a grid will benefit from having storage technologies with a range of durations so that some may be used to meet peak loads (cycling frequently) while others provide power during extended times of poor weather (cycling infrequently).

[1]Environmental Systems Graduate Program, School of Engineering, University of California Merced, Merced, CA 95343, USA

[2]Goldman School of Public Policy, University of California, Berkeley, 450 Sutardja Dai Hall, Berkeley, CA 94720, USA

[3]Mechanical Engineering Graduate Program, School of Engineering, University of California Merced, Merced, CA 95343, USA

[4]Aerospace Engineering Department, Faculty of Engineering, Cairo University, Giza 12613, Egypt

[5]Lead contact

*Correspondence: zabirsami@gmail.com

https://doi.org/10.1016/j.isci.2022.105900

This is an open access article under the CC BY license (http://creativecommons.org/licenses/by/4.0/).

Some studies have estimated the annual number of cycles for different energy storage applications, including "arbitrage" (when the storage is charged at a time of low prices and discharged at a time of high prices) and "seasonal" (when the storage is only charged and discharged seasonally). For storage applications labeled as "arbitrage," the cycles per year have been identified as 50-400,[16] 250 +[17], 300-400,[18] and 90-365.[19] For storage applications labeled as "seasonal," this number has been reported as 1-5 cycles per year.[16,19]

State-of-the-art energy systems modeling today often uses capacity-expansion models that identify the lowest cost workable solution based on projected costs of a set of competing technologies.[20–24] Such a modeling approach is best when the projected costs are well known, and the purpose of the modeling is to find the economic technology mix and the optimal design of the given assets. Alternatively, it is also common practice to apply policy constraints that lead to a given technology mix and optimize the other technologies around those.

While the current tools select the lowest cost way to implement a given set of candidate storage technologies, the focus is usually on the selected solutions, which can be highly variable in a vast parameter space. What we seek is a complementary tool that answers questions like: If I make a 12-h battery rather than an 8-h battery, how frequently will I use the additional 4 h of capacity? While there is still a large parameter space to be explored, the causality between the generation-load profiles and needed storage can be explored more transparently, enabling the development of an intuition about the causality, which can then inform the use of other tools.

In this article, we propose a novel hierarchical method that estimates the minimum requirements for the amount and frequency of use of energy storage for a given set of load and generation profiles. The hierarchical method defines a set of storage assets that are filled and emptied according to a hierarchy,(see Method Details section and associated figures) much like the lowest cost generators bid into a market are selected before higher cost generators. We fill and empty these bins according to the hierarchyand document the number of times they are cycled over a year to differentiate the multiple uses of the storage. To demonstrate the value of the method, we then define a set of scenarios and apply the method for each, discussing how the method enables us to differentiate the types of storage that would be needed under the different scenarios. Finally, we discuss how the hierarchical storage method provides us with information that is not readily available from other approaches as well as providing intuition for more detailed modeling and talk about how our results relate to those of other studies. Many current energy storage studies focus on the *duration* of energy storage. However, in our work, we concentrate on the *frequency* of cycling statistics of energy storage. This approach complements the existing studies based on energy-to-power rating by identifying how frequently we would call upon a shorter- or longer-duration storage asset.

RESULTS AND DISCUSSION

In Figure 1, we plot the number of complete cycles each storage bin experiences as a function of the bin hierarchy, with the highest priority bin shown as the smallest value on the x axis. Instead of labeling the x axis with the bin number, we use units that reflect the fraction of the total annual load. Figure 1 shows that the highest priority bin (first used bin) is cycled essentially every day (about 360 times/year - top left of the graph) while between 3% (6.5TWh) and 9% (19TWh) of the electricity delivered each year (lower right of the graph) would be put into a storage asset that is cycled only once per year. The graph in Figure 1 uses log-log axes to capture the detail of the charging of the first bins and the large amount of energy required to meet the seasonal needs. Traces are plotted for eight scenarios, replacing some of the scaled-up historical solar with the alternative wind or simulated solar generation profiles described in Table 1.

The first thing to note in Figure 1 is that the curves appear to fall into natural groupings with a horizontal section in the upper left, a horizontal section in the lower right, and a diagonal or vertical (irregular) section linking the two horizontal sections. We have added a horizontal line indicating when the storage is cycled once every two days (at 183 cycles/year) and a second horizontal line for when the storage is cycled twice per year. These lines indicate somewhat natural breaks in the data and facilitate our discussion of the results. The data above the top line are summarized in the first part of Figure 2 and will be referred to as diurnal storage because of being used on a daily or almost daily basis. The data below the bottom line

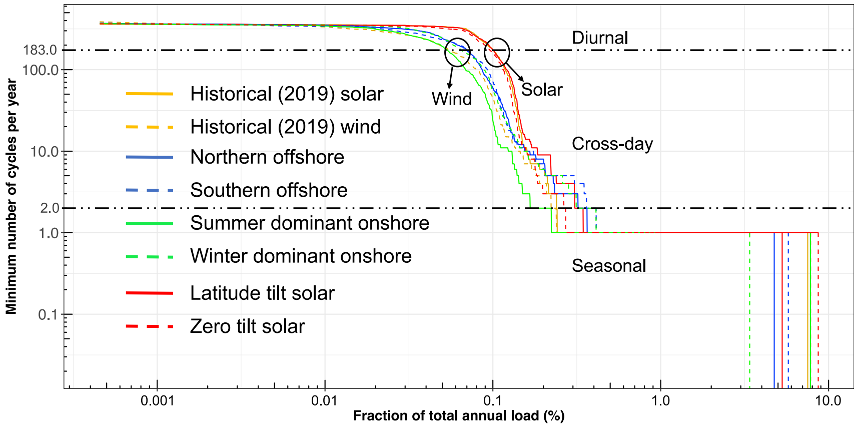

Figure 1. Minimum number of cycles as a function of storage amount expressed in terms of the fraction of total annual load using log-log axes

Horizontal dashed lines for the number of cycles = 183 (indicating cycling on average of 1 ×/2 days) and = 2 (indicating cycling 2 ×/year) are drawn to differentiate types of storage applications. Two circles highlight the difference between the added-solar and added-wind scenarios. For each scenario, 40% of the total annual load is from the 2019 historical generation. Energy mix used for the other 80%: added-solar cases = 80% from 2019/latitude/zero tilt solar and added-wind cases = 60% from 2019 solar and 20% from the indicated wind generation profile.

are summarized in Figure 2 as seasonal storage, and the data between the two lines are summarized as cross-day storage.

The minimum number of cycles per year shown in Figure 1 is an essential metric for several reasons. It (shown on the y axis) indicates the income that may be expected from selling the generated electricity. The frequency of cycling will also contribute to the degradation of storage assets, especially those that measure lifetime by the number of cycles that can be completed. The x axis shows the storage amount as a fraction of the annual load, giving an idea of the ratio of total storage required to the total annual load, including the amount of storage that may be expected to be cycled frequently and the amount that may be cycled infrequently. We emphasize that the shown usage is a minimum requirement calculated for a system with perfect transmission. A real system will experience congestion, benefiting from additional local storage, thereby increasing the frequency of use.

For all eight scenarios, the bins that cycle every day or two require a capacity to hold 0.1% of the annual load. There is very little difference between the three solar-rich scenarios in the curves at the top of the graph. The solar-rich scenarios all show a rapid drop in the frequency of use once the hierarchy passes

Table 1. Description of the renewable electricity generators with their modeling parameters

Energy resource	Number of modeled sites	Modeling assumptions
Solar PV		
Historical (2019)	CAISO data	Module type: Standard, Array type: 1-Axis tracking, Total system losses: 14.08%, DC to AC ratio: 1.2, Data collected: 2012, Data type: hourly
Latitude tilt	3	
Zero tilt	3	
Onshore Wind		
Historical (2019)	CAISO data	Turbine power curve: GE 2.5-120, Hub height: 100m, Data collected: 2012, Data type: 5-min interval
Summer dominant	4	
Winter dominant	5	
Offshore Wind		
Northern	3	Turbine power curve: Aerodyn SCD 8.0/168, Hub height: 120m, Data collected: 2019, Data type: 5-min interval
Southern	2	

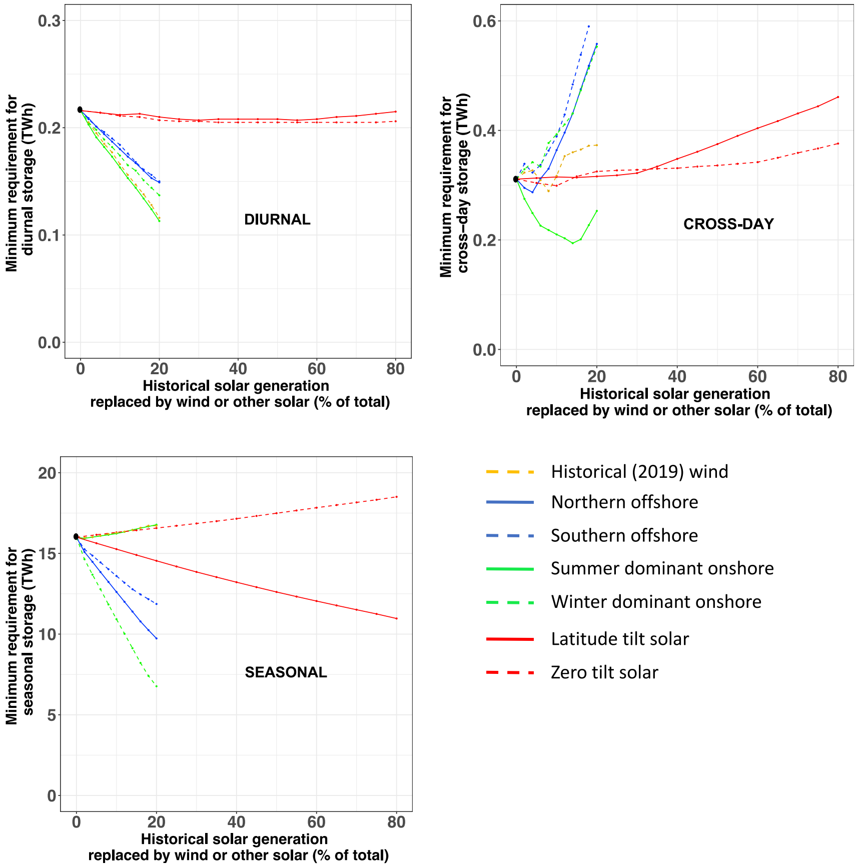

Figure 2. Variations in the minimum required size of diurnal, cross-day, and seasonal energy storage for eight scenarios as a function of the fraction of the historical solar generation that was replaced by one of the wind- or other-solar-generation profiles

Solar scenarios are extended to 80% and wind scenarios are up to 20% reflecting the relative availability of solar and wind. The black dots represent the baseline scenario results.

about 0.1% of annual load, quickly shifting from using the storage almost every day to using the storage only once per year. This change from frequent to infrequent cycling reflects the shift from needing to supply electricity at night to needing to supply electricity during the winter (or during a severe heat event in late summer). However, despite the relatively fast drop of the solar curves for the log-log scale used in Figure 1, the amount of storage needed for a series of cloudy days (0.2-0.6 TWh) is comparable to that needed to get through the night (0.1-0.2 TWh), as can be seen more clearly in Figure 2. The three solar-rich scenarios diverge more markedly when one considers the amount of storage that will cycle just once per year as shown in both Figure 1 and in Figure 2. The latter figure uses a linear instead of a logarithmic scale, so it is easier to interpret quantitatively and is discussed below.

Considering the five scenarios in Figure 1 that have added wind, we see that far less storage is frequently cycled compared with the solar-rich scenarios. This difference is highlighted by the two black circles in Figure 1 and by the strong differentiation of the curves in Figure 2. This is especially true for the summer-dominant onshore wind that typically shows peak generation at night. There are also significant differences in the amount of storage that these five scenarios require to get through the winter. As shown by the overlapping lines in Figure 1, the result for the historical wind generation profile is somewhat similar to the summer-dominant onshore wind in terms of diurnal storage and the overall amount of energy storage needed. We note that, although we have modeled this storage here as electricity converted to some other form of energy and then later converted back to electricity, there is likelihood that this seasonal need for storage could be met with hydrogen made without carbon dioxide emission or that the seasonal challenge

may be met by building more solar and wind that are either curtailed during times of over-generation or used for generating hydrogen or some other valuable product. Thus, we present this analysis as a way of assessing the size of the need rather than suggesting a solution.

The effects of the eight scenarios on the needed diurnal/cross-day and seasonal storage directly reflect the daily and seasonal correlation between supply and demand and increase monotonically as the baseline solar generation is replaced with an alternative generation. As can be seen from Figures 1 and 2, all of the solar-rich scenarios show consistent use of diurnal storage while the addition of wind reduces the need for diurnal storage with the historical and summer-dominant onshore wind having the most significant effect and the offshore wind having the smallest (yet substantial) effect. The effect of the eight scenarios on the seasonal mismatch is also substantial with the use of winter-dominant onshore wind reducing the size of the seasonal mismatch by almost a factor of two, the two offshore wind scenarios reducing it by about half that, and the effect of the solar tilt either increasing the seasonal need (when the zero tilt is optimized to increase summer generation) or decreasing it (when a south-facing tilt increases winter generation). The calculated cross-day storage found for the eight scenarios depends more on the level of replacement of the alternative generation technology. The offshore wind and winter-dominant onshore wind show the greatest need for cross-day storage. In contrast, the summer-dominant onshore wind, which is largely driven by a diurnal sea breeze, decreases the need for cross-day storage, though larger amounts reverse that effect. The observed increase in cross-day storage required for high levels of the simulated latitude- and zero-tilt solar may be an artifact of the limited area sampled by those solar profiles.

It can easily be seen from Figure 2 that replacing solar generation with wind generation reduces the diurnal storage but increases the cross-day storage by a similar amount. Thus, adding wind generation effectively decreases the cycling frequency while still needing a storage fleet with a similar energy capacity when the diurnal storage and cross-day storage are considered together. Using the tilt of the solar panel or selecting a different wind resource has some effect on the amount of diurnal and cross-day storage needed, but these effects are much smaller than the difference between solar and wind scenarios. The biggest difference between different types of solar and wind resources is clearly seen in Figure 2 when comparing the seasonal storage that is needed. The summer-dominant and winter-dominant wind diverge quite markedly with the offshore wind having a smaller effect but generally helping to decrease the need for seasonal storage. We see an increasing need for seasonal storage when we increase the summer-dominant wind fraction in the energy mix. However, for winter-dominant onshore wind addition, the required size of seasonal storage decreases by more than a factor of two with a replacement of just 20%. Similarly, the need for seasonal storage decreases by about 40% while shifting from zero-tilt to latitude-tilt solar.

From Figures 1 and 2, we can see how the hierarchical method provides a straightforward and clear way to identify the natural differentiation of different uses of the storage, quantifying how the needs for diurnal, cross-day, and seasonal storage vary with the chosen generation mix. The value of the hierarchical method is demonstrated by this discussion, which would be much more difficult to derive from more conventional capacity-expansion modeling. The hierarchical method can also answer other questions, such as whether the required discharge power varies for the storage that is cycled frequently and infrequently.

Statistics of discharging rates are shown in Figure 3 for the storage that is cycled most frequently (on the left) to the storage that is cycled least frequently (on the right). Each type of storage may be sized in power capacity to deliver the maximum demand required of it. For the most frequently cycled storage (diurnal), the maximum discharge rate is observed to be 35 GW with the most frequently observed discharge powers in the range of 13-22 GW. For the less frequently cycled storage (seasonal), the maximum discharge rate is observed to be smaller (28 GW), and the distribution of discharge powers observed tends to be much tighter, typically about 18-20 GW. The cross-day storage shows the narrowest peak for distribution of discharge power at 18-19 GW while the maximum discharge rate tends to be around 25 GW. Figures 1-3 combined to demonstrate how the hierarchical method can elucidate the types of storage that will be required as a minimum for projected scenarios.

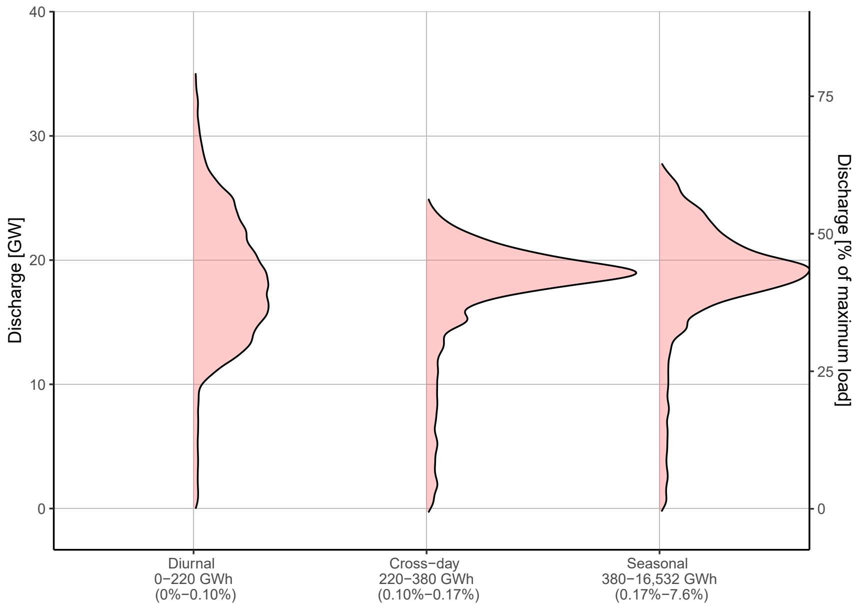

Figure 3. Frequency distribution of discharge rates for diurnal (0-220 GWh), cross-day (220-380 GWh), and seasonal (380-16,532 GWh) storage bins for the baseline scenario

Fraction of the annual load is shown in the parentheses along the x axis. Both discharge in GW (left) and discharge as a fraction of maximum load (right) are represented along y axis.

As we are not certain about which technology will be primarily used in case of different types of storage needs, we analyze how assumptions on round trip efficiency could affect relative needs of storage as shown in Figure 4. We find that decreasing round-trip efficiency shifts the curves slightly to the right but does not change the overall shape of the curves. In Figure 4 we compare scenarios that replace a greater fraction of the solar with wind, which may be implausible for California, but is relevant for regions with stronger wind resources. We see that scaling up the wind to generate 80% of the annual load decreases the diurnal storage requirement by a larger amount. This is because of the generation profile of wind having higher electricity production during the nighttime unlike solar.[25]

The hierarchical approach to tracking energy use may appear to be an academic exercise, but it can be a realistic representation of the use of storage in a competitive market. Wholesale markets use a bidding process to select the lowest-cost generators. Storage with low upfront and operating costs, high efficiency, and long lifetime bid into the market at the lowest prices. Thus, the market will select to charge and discharge the storage assets in a hierarchical way giving priority to those with the lowest bids. While we could treat the storage as one large hypothetical storage reservoir, the hierarchical approach allows analysis of the frequency of cycling at a more detailed level.

The cycling frequencies shown in Figures 1 and 4 provide a clear guide for the types of storage that will be needed for the given generation and load profiles, assuming no congestion effects. Using the simplistic assumption of constant load throughout the year, the "fraction of total annual load" axis can be used to estimate the number of hours a storage asset would be able to discharge. For the solar-dominant scenarios, Figure 1 shows cycling of greater than 183 times/y (more than once every two days) for storage of a size that could meet up to 0.1% of annual load. This storage would be able to provide power to meet the average load for 8-9 h. Thus, storage with a rated duration of 8-9 h is expected to be used mostly for diurnal applications.

For the scenarios shown in Figure 1, any storage asset with greater than 24 h of capacity would be expected to only be cycled fully once per year, while the "wind only" scenario in Figure 4 would use a 24-h storage asset more frequently. For the "wind only" scenario, about 1.1 TWh of storage cycles between 2 and 183 times annually while for the only solar scenario, it is only 0.33 TWh storage having the same number of

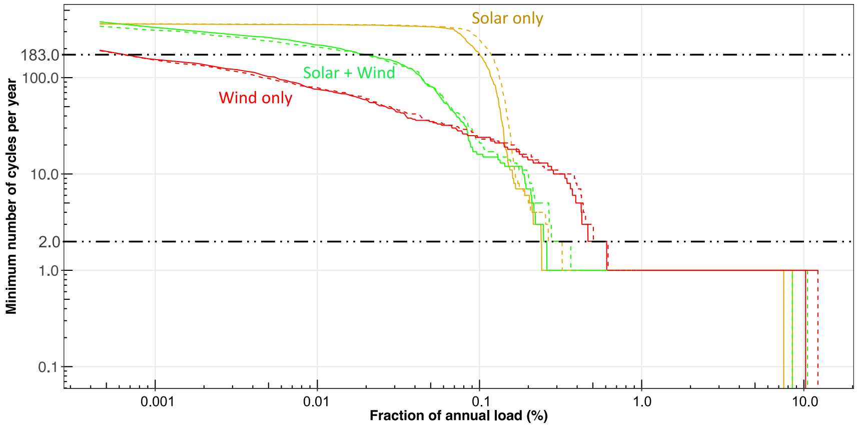

Figure 4. Analysis of sensitivity to round trip efficiencies

Solid lines indicate 80% round trip efficiency and dashed lines represent 50%. Total generation for all three cases equals 120% of total annual load. 40% of generation consists of 2019 historical generation. Rest 80% are as follows: all from 2019 solar (solar only), half from 2019 solar and half from 2019 wind (solar - wind), all from 2019 wind (wind only).

cycles. While there can be a debate about the number of hours that differentiates energy storage technologies, our approach provides data that can be helpful in evaluating the frequency of cycling that may be expected for a storage asset of different sizes. We emphasize that the results of the method are highly dependent on the generation and load profiles that are put into it, so the results here may not be relevant to other generation and load profiles.

The application of the results may be confusing to assess, so we give a couple of examples here. For a solar-dominated grid, if a storage product is offered that can bid into the market competitively relative to all other storage assets, then it can count on cycling almost every day or at least every other day up to nearly 8 h of storage. If the energy reservoir on that product is expanded to hold 12 h of storage and the asset is still bid into the market at the most competitive price, the additional 4 h of capacity is likely to be used less frequently with the full energy range used approximately once a week. These numbers will increase if there are local congestion issues, which are common. There might be congestion issues while transmitting electricity that can be resolved by adding storage in the congested areas. Congestion issues can be included in our method if location-specific generation and load profiles are used.

As a second case study, we consider a storage product that has a lower efficiency (or some other reason for bidding into the market at a higher price). The position in the hierarchy may not be consistently defined, but we take as an example a 50%-efficient storage asset that is otherwise identical to a 90%-efficient asset. In this case, the 50% asset will need to be bid into the market at a higher price to account for the need to charge almost twice as much electricity as the 90% asset. In such a case, the 50% asset may find it difficult to access the diurnal application and may be limited to successfully bidding in the market when 90% of assets have been fully discharged, decreasing its discharge frequency. On the other hand, the income per cycle associated with arbitrage is likely to increase as one moves to the right in Figure 1, potentially compensating for the small number of cycles. Thus, our method is most helpful in identifying how the needed storage assets will change for a range of generation and load profiles and less useful in quantifying the income associated with each cycle. In practice, many things will determine the hierarchy, including idle losses and degradation rates. The curves shown in Figure 1 help to quantify the expected use frequency that may be anticipated based on the number of hours of energy storage an asset can store, enabling the needed cycle life and assessing its ability to compete with other storage technologies.

This article demonstrates how the hierarchical approach can define minimum storage requirements for a given set of generation and load profiles, providing quantitative information about cycling frequencies, amount of storage needed for frequently and infrequently cycled applications, length of time energy is stored, and the discharge powers that may be needed in different situations. In practice, congestion may result in more frequent cycling and/or the need for more storage for the defined generation- and load-profile pair. Although we do not expect our results to exactly duplicate those found by

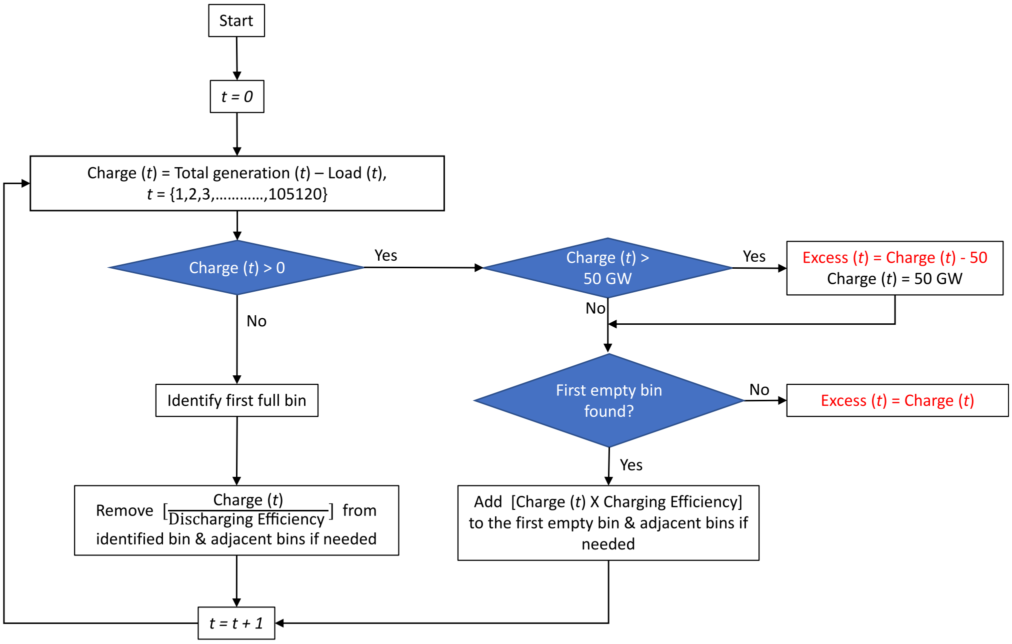

Figure 5. Flowchart of our hierarchical approach's working mechanism
Dis/charging efficiency is assumed 89.44% resulting in 80% round-trip efficiency.

complementary studies, it is useful to compare our results with other published results. A study shows the installed energy storage capacity will be around 7% of the total generation capacity in the Western US Power System,[20] consistent with the 5%-11% found for the wide range of scenarios we show in Figures 1 and 4 using a far simpler approach.

While our approach (as shown in Figures 5, 6, and 7) has assumed that all differences between generation and load will be accommodated by using our hypothetical hierarchical storage, we anticipate that it will be most economical to meet the needed seasonal storage by using cross-sector approaches such as the storage of hydrogen that is used for powering the transportation and industrial sectors. Thus, our calculation identifies the size of the seasonal challenge but does not pretend to describe the solution to that challenge.

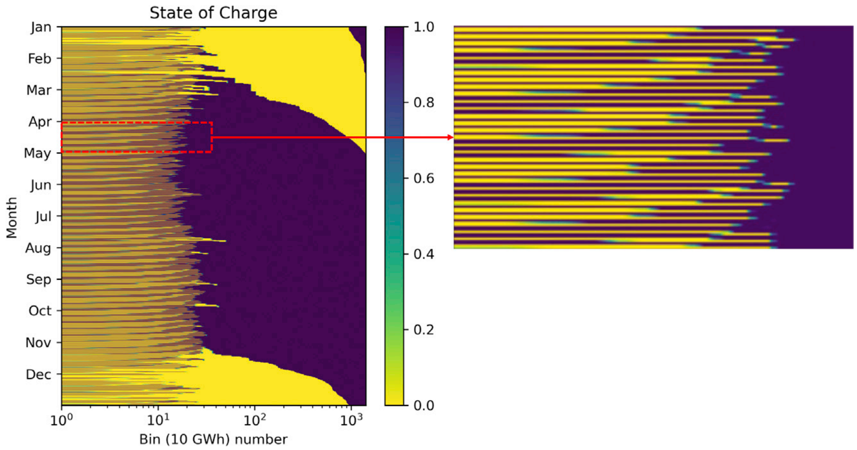

Figure 6. State of charge of each storage bin using the hierarchical approach
Figure on right represents daily charging and discharging of bins.

CellPress
OPEN ACCESS

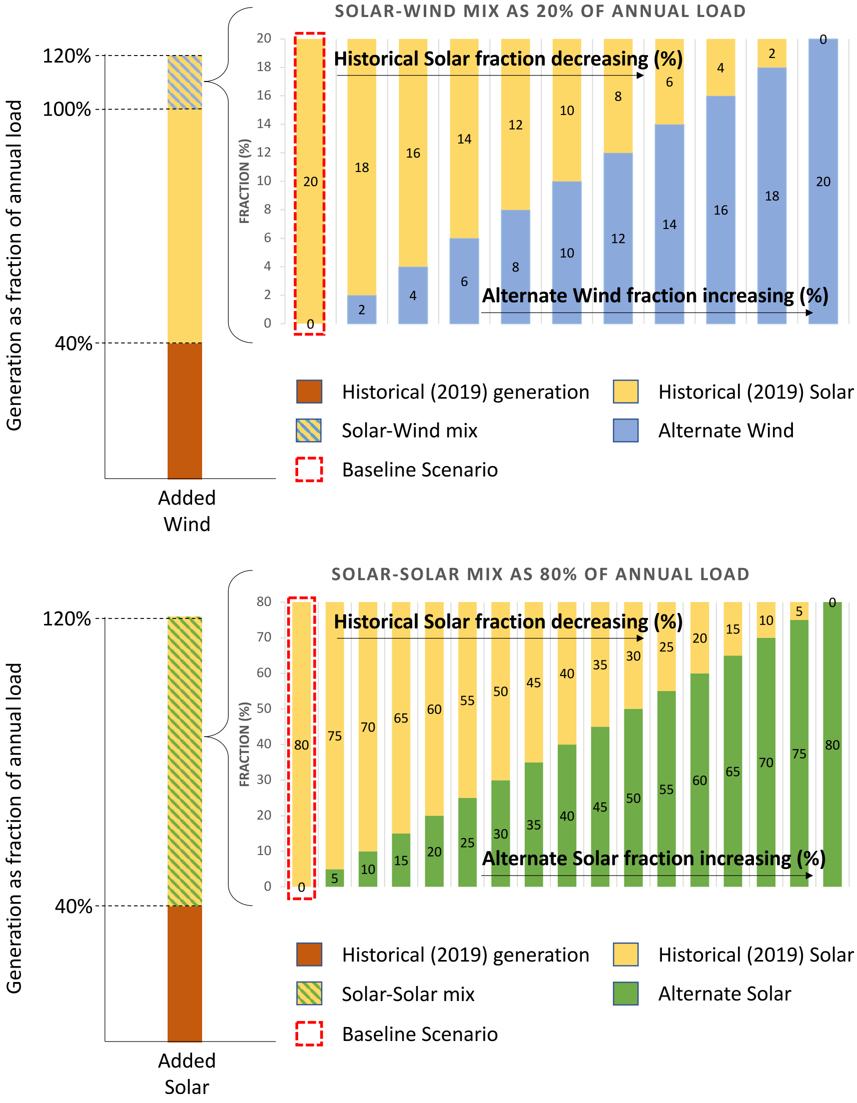

Figure 7. Energy generation mix for different scenarios

For added wind scenarios, in the top right graph, the first step uses 20% solar and 0% wind while the last step takes 20% wind and 0% solar. In each step, wind increases and solar decreases by 2% to make the total amount 20% in every step. For added solar scenarios, the bottom right graph shows how the historical solar decreases and alternate solar increases by 5% in each step resulting in 80% of the total annual load. Energy mix marked by a red dashed line represents the baseline scenario.

Conclusion

As governments and companies develop the storage that will be used to support renewable energy-driven grids, questions arise about how frequently the storage may be cycled, how long it may need to hold a charge between cycles, and the related question of what duration of storage will be needed. Here, we have described and demonstrated a new hierarchical method for quantifying the cycling frequency and minimum amount (both in terms of the energy and power ratings) of storage needed for projected generation and load profiles. The demonstration of the method shows how a solar-driven grid requires a) diurnal storage to get through the night, b) a comparable amount of storage that will be cycled less frequently, and c) a much larger amount of seasonal storage that is cycled once or twice per year. Although we have studied profiles relevant to California, solar-dominant grids for all sunny locations will have fairly similar diurnal requirements, while the latitude will affect the seasonal requirements and cloudy weather patterns will

affect the cross-day requirements. A grid that is driven by a mixture of wind more than solar needs roughly the same amount of storage but cycles that storage less frequently. The reduced cycling may reduce degradation but also reduces the anticipated revenue from selling the discharged electricity, suggesting that such storage technologies will either need to be lower in cost or use market structures that compensate for providing reserve power. Moreover, the results showed that latitude-tilt solar and wind that generates more during the winter could greatly reduce the seasonal storage requirement. The highest power rating is needed for the storage that is cycled most frequently, but the seasonal storage may be required to discharge at a power approaching the highest, especially if the electricity demand during the winter increases relative to the 2019 demand curve used in this study.

The hierarchical method complements more detailed capacity expansion modeling by providing a simple way to guide investment in new storage technologies as well as the ability to explore a wide range of scenarios to gain intuition about how choices such as investing in wind versus solar or latitude-tilt solar versus zero-tilt solar can affect the storage requirement. Similarly, the method can gain intuition about how modifications to the load profile, perhaps by shifting flexible loads, can affect the need for storage as well as clarify the discharge power requirements for frequently and infrequently cycled storage. The method may be applied to a small or a large grid, but the assumption of perfect transmission availability will limit its value for evaluating large grids. The assumption of hierarchical usage of the storage may not apply if the same storage products are used for daily cycling and for retaining charge over weeks or months. Nevertheless, it is useful to understand how a system using a portfolio of technologies might be most efficiently used by dis/charging the assets that can bid at the lowest cost on a daily basis, while using less efficient but very inexpensive storage technologies for resource adequacy over weeks and months. This hierarchical strategy complements grid expansion plans that minimize the cost of delivering adequate electricity by helping to identify the best candidate technologies to model in the cost optimization. The method may be extended to consider the cycling frequency that would be expected when grid congestion is considered and applied to the outputs of capacity expansion models to better understand how storage is being used. While capacity expansion models are designed to identify the lowest cost way of meeting electricity demand, our method may help to identify what candidate storage technologies will be most attractive to the model and provide a quick calculation that can explore a broader range of parameter space.

Limitations of the study

The method does not consider the profitability of storage or the market investment in storage, as this requires simulating prices using detailed capacity-expansion models. It complements these models by providing insight into how often storage needs to cycle without making assumptions about price or lifetime. The cost of storage technologies will influence market prices and hence the need for storage and the load profile are interdependent.

Additionally, this approach doesn't include the following.

1. Considering transmission losses
2. Modifying hydro generation by scaling up/down to balance load and generation
3. Adjustment in load profiles for future heat pump and EV adoption and demand management

STAR★METHODS

Detailed methods are provided in the online version of this paper and include the following:

- KEY RESOURCES TABLE
- RESOURCE AVAILABILITY
  - Lead contact
  - Materials availability
  - Data and code availability
- METHOD DETAILS

SUPPLEMENTAL INFORMATION

Supplemental information can be found online at https://doi.org/10.1016/j.isci.2022.105900.

ACKNOWLEDGMENTS

This document was prepared as a result of work sponsored by the California Energy Commission. It does not necessarily represent the views of the Energy Commission, its employees, or the State of California. The Energy Commission, the State of California, its employees, contractors, and subcontractors make no warranty, express or implied, and assume no legal liability for the information in this document; nor does any party represent that the use of this information will not infringe upon privately owned rights. This report has not been approved or disapproved by the Energy Commission nor has the Energy Commission passed upon the accuracy of the information in this report.

AUTHOR CONTRIBUTIONS

Z. M.: methodology, software, formal analysis, visualization, and writing. K. S.: visualization, writing – review & editing. M. A.: software, writing – review & editing. P. S.: software, writing – review & editing. S. K.: conceptualization, project administration, writing – review and editing, supervision, formal analysis, and funding acquisition.

DECLARATION OF INTERESTS

The authors declare no competing interests.

INCLUSION AND DIVERSITY

One or more of the authors of this article self-identifies as an underrepresented ethnic minority in science.

Received: August 17, 2022
Revised: October 26, 2022
Accepted: December 23, 2022
Published: January 20, 2023

REFERENCES

1. International Energy Agency (IEA) (2020). Renewable energy market update: Outlook for 2020 and 2021. https://www.iea.org/reports/renewable-energy-market-update.

2. International Renewable Energy Agency (IRENA) (2020). Global renewables outlook. https://www.iea.org/reports/world-energy-outlook-2020.

3. Pörtner, H., Roberts, D., Adams, H., Adler, C., Aldunce, P., Ali, E., Begum, R., Betts, R., Kerr, R., Biesbroek, R., and Birkmann, J. (2022). Climate change 2022: Impacts, adaptation and vulnerability. IPCC sixth assessment report.

4. Koohi-Fayegh, S., and Rosen, M.A. (2020). A review of energy storage types, applications and recent developments. J. Energy Storage *27*, 101047.

5. Mongird, K., Viswanathan, V., Balducci, P., Alam, M., Fotedar, V., Koritarov, V., and Hadjerioua, B. (2019). Energy storage technology and cost characterization report. https://www.osti.gov/servlets/purl/1573487.

6. Shan, R., Reagan, J., Castellanos, S., Kurtz, S., and Kittner, N. (2022). Evaluating emerging long-duration energy storage technologies. Renew. Sustain. Energy Rev. *159*, 112240.

7. Luo, X., Wang, J., Dooner, M., and Clarke, J. (2015). Overview of current development in electrical energy storage technologies and the application potential in power system operation. Appl. Energy *137*, 511–536.

8. Hunt, J.D., Byers, E., Wada, Y., Parkinson, S., Gernaat, D.E.H.J., Langan, S., van Vuuren, D.P., and Riahi, K. (2020). Global resource potential of seasonal pumped hydropower storage for energy and water storage. Nat. Commun. *11*, 947.

9. Stocks, M., Stocks, R., Lu, B., Cheng, C., and Blakers, A. (2021). Global atlas of closed-loop pumped hydro energy storage. Joule *5*, 270–284.

10. Ziegler, M.S., and Trancik, J.E. (2021). Re-examining rates of lithium-ion battery technology improvement and cost decline. Energy Environ. Sci. *14*, 1635–1651.

11. US Energy Information Administration (EIA) (2020). Battery storage in the United States: an update on market trends. https://www.eia.gov/analysis/studies/electricity/batterystorage/pdf/battery_storage_2021.pdf.

12. Hu, X., Xu, L., Lin, X., and Pecht, M. (2020). Battery lifetime prognostics. Joule *4*, 310–346.

13. Kittner, N., Castellanos, S., Hidalgo-Gonzalez, P., Kammen, D.M., and Kurtz, S. (2021). Cross- sector storage and modeling needed for deep decarbonization. Joule *5*, 2529–2534.

14. Van der Linden, S. (2006). Bulk energy storage potential in the USA, current developments and future prospects. Energy *31*, 3446–3457.

15. Benitez, L.E., Benitez, P.C., and van Kooten, G.C. (2008). The economics of wind power with energy storage. Energy Econ. *30*, 1973–1989.

16. Schmidt, O., Melchior, S., Hawkes, A., and Staffell, I. (2019). Projecting the future levelized cost of electricity storage technologies. Joule *3*, 81–100.

17. Akhil, A., Huff, G., Currier, A., Kaun, B., Rastler, D., Chen, S., and Cotter, A. (2013). Doe/epri 2013 electricity storage handbook in collaboration with nreca. https://www.sandia.gov/ess-ssl/publications/SAND2015-4982.pdf.

18. Rastler, D.M. (2010). Electric energy storage technology options: a white paper primer on applications, costs and benefits. http://large.stanford.edu/courses/2012/ph240/doshay1/docs/EPRI.pdf.

19. International Energy Agency (IEA) (2014). Technology roadmap - energy storage. https://www.iea.org/reports/technology-roadmap-energy-storage.

20. Guerra, O.J., Zhang, J., Eichman, J., Denholm, P., Kurtz, J., and Hodge, B.M. (2020). The value of seasonal energy storage technologies for the integration of wind and solar power. Energy Environ. Sci. *13*, 1909–1922. https://doi.org/10.1039/D0EE00771D.

21. Sepulveda N.A., Jenkins J.D., Edington, A., Mallapragada D.S., and Lester R.K.. (n.d.). The design space for long-duration energy

storage in decarbonized power systems. Nat. Energy, 6, 506-516.

22. Sánchez-Pérez, P., Staadecker, M., Szinai, J., Kurtz, S., and Hidalgo-Gonzalez, P. (2022). Effect of modeled time horizon on quantifying the need for long duration storage. Appl. Energy *317*, 119022.

23. Nelson, J., Johnston, J., Mileva, A., Fripp, M., Hoffman, I., Petros-Good, A., Blanco, C., and Kammen, D.M. (2012). High-resolution modeling of the western north american power system demonstrates low-cost and low-carbon futures. Energy Pol. *43*, 436–447.

24. Mileva, A., Johnston, J., Nelson, J.H., and Kammen, D.M. (2016). Power system balancing for deep decarbonization of the electricity sector. Appl. Energy *162*, 1001–1009.

25. California independent system operator (CAISO) (2021b). Managing oversupply. http://www.caiso.com/informed/Pages/ManagingOversupply.aspx.

26. California Public Utility Commission (2019). Inputs & assumptions: 2019-2020 integrated resource planning. https://www.cpuc.ca.gov/-/media/cpuc-website/divisions/energy-division/documents/integrated-resource-plan-and-long-term-procurement-plan-irp-ltpp/2019-2020-irp-events-and-materials/inputs-assumptions-2019-2020-cpuc-irp_20191106.pdf.

27. Mahmud, Z., Shiraishi, K., Abido, M.Y., Millstein, D., Sánchez-Pérez, P.A., and Kurtz, S. (2022). Geographical variability of summer- and winter-dominant onshore wind. J. Renew. Sustain. Energy *14*, 023303.

28. Energy and Environmental Economics, Inc. (E3) (2021). Resolve: renewable energy solutions model. https://www.ethree.com/tools/resolve-renewable-energy-solutions-model/.

29. Energy Information Agency. (EIA) (2018). Form EIA-923 detailed data with previous form data (EIA-906/920). https://www.eia.gov/electricity/data/eia923/.

30. Draxl, C., Clifton, A., Hodge, B.-M., and McCaa, J. (2015). The wind integration national dataset (WIND) toolkit. Appl. Energy *151*, 355–366. https://doi.org/10.1016/j.apenergy.2015.03.121.

31. Dobos, A. (2014). Pvwatts Version 5 Manual (No. Nrel/tp-6a20–62641). https://pvwatts.nrel.gov/.

CellPress
OPEN ACCESS

STAR★METHODS

KEY RESOURCES TABLE

REAGENT or RESOURCE	SOURCE	IDENTIFIER
Deposited Data		
California Independent System Operator (CAISO) generation and demand data	CAISO	http://www.caiso.com/informed/Pages/ManagingOversupply.aspx
Wind speed and wind output	National Renewable Energy Laboratory (NREL) WIND Toolkit	https://maps.nrel.gov/wind-prospector
Solar irradiance	National Solar Radiation Database (NSRDB) Data Viewer	https://nsrdb.nrel.gov/data-viewer
Solar output	PVWatts Calculator	https://pvwatts.nrel.gov/

RESOURCE AVAILABILITY

Lead contact

Further information and requests for resources and reagents should be directed to and will be fulfilled by the lead contact, Zabir Mahmud (zabirsami@gmail.com).

Materials availability

This study did not generate any new unique reagents.

Data and code availability

- This paper analyzes existing, publicly available data which are listed in the key resources table.
- Code for the energy balance approach was written in Python and is available from the lead contact upon request
- Any additional information required to reanalyze the data reported in this paper is available from the lead contact upon request

METHOD DETAILS

Our hierarchical method balances the electricity production and demand by charging and discharging a hypothetical set of storage bins. Charge is calculated for each time step, t, by the difference between total generation and load as shown in Figure 5. A 50-GW charging rate limit is applied to reflect a charge rate commonly observed without suggesting investment in hardware that would be required infrequently, with the unused electricity labeled as "Excess" in Figure 5. The 50-GW limit was chosen by examining the frequency distribution observed if we did not place the limit. This Excess electricity may be curtailed, but it might also be used for another purpose. We create a set of 10-GWh storage bins and charge and discharge them according to a predefined hierarchy. The bins are charged and discharged based on the calculated energy balance, including dis/charging efficiency losses, as noted in Figure 5. The algorithm forces the state of charge on January 1 to equal that at the end of December 31 by limiting the number of bins and documenting additional surplus electricity as "Excess." We use a "first bin first" approach for charging and discharging the bins. While charging, for each time step, the algorithm starts to check from the first bin. If there is capacity available, the charge is placed in that bin. If not all of the charge can be accommodated in the first bin, the algorithm goes on to the next until all of the charge has been placed in a bin. Similarly, during discharging, the model searches for energy to provide from the first bin, and if we need more than that bin is holding, the model goes to the next one.

We record how many times each bin is filled and emptied, then summarize the number of cycles per year for each bin as the primary output of the method. Figure 6 is a visual representation of the bins filling and emptying throughout the year. A logarithmic scale is used on the x axis of Figure 6 to highlight the daily use of the first storage bins and the seasonal use of all of the storage. A section of the left graph in Figure 6

is expanded on the right in the same figure to see the filling and emptying processes in more detail. One month's charging and discharging of the bins is represented in the right figure, whereas the left one demonstrates the filling and emptying of bins throughout one year.

For discussion purposes, we define three types of energy storage based on the number of times the storage is cycled per year: diurnal storage, cross-day storage, and seasonal storage. We define diurnal storage that charges and discharges completely at least once every two days (i.e., greater than 183 cycles per year) on average over a year. We define seasonal storage as any storage bins that cycle 1–2 times a year. When the number of cycles is between 2 and 183 per year for any bins, we define those bins as cross-day storage for discussion purposes but note that the user of the method may define different categories, especially if the analysis finds different natural groupings.

We examine the need for energy storage by balancing the load and generation using 5-min temporal resolution for renewable energy portfolios with different characteristic generation profiles in California. These generation profile data and documented load profiles provide a strategy for simulating a range of plausible future renewable-electricity grid scenarios. We selected California because of the availability of data documenting relatively high wind (7%) and solar (13%) utility-scale generation. Results from this analysis are site-specific as load and generation profiles can vary between regions. However, this method is applicable to any location throughout the world. Still, it is most relevant for small regions because it assumes consistent availability of transmission within the load-generation zone and, therefore, provides a lower bound on the energy storage requirements. To demonstrate the value of our new method, we design eight renewable energy portfolios (as listed in the first column of Table 1) to support a 100% zero carbon grid in California assuming perfect transmission capability. We scale up the 2019 5-min-resolution solar- and wind-generation profiles reported by CAISO[25] to create generation mixes that could be plausible in future years depending on the relative growths of solar, onshore wind, and offshore wind. We also use the 2019 load profile data from CAISO, neglecting anticipated changes in the load over the coming years, to demonstrate the method and elucidate trends without attempting to model the future accurately. As the load and generation profiles might vary for different years, the minimum storage requirements can potentially change. For simplicity, we assume that a significant increase in solar and/or wind generation will entirely replace the current fossil-fired, nuclear, and imported power. To focus on the study objectives, the generation from biomass, geothermal, small and large hydropower is assumed unchanged.

In our baseline scenario (marked by red dashed rectangular region in Figure 7), we assume that all additional generation comes from solar with no change in the 2019 solar power generation profile as the solar generation is scaled up to not only replace the fossil-fuel, nuclear, and imported electricity, but to add excess generation so that the total generation during the year is 120% of the annual load after considering the losses associated with inefficiency of the dis/charging of the storage. The eight scenarios considered in this analysis could be divided into two types: added solar and added wind. For both, generation equal to 40% of the total annual load (bottom part of left two bar graphs in Figure 7) comes from the retained historical generation that includes solar, wind, biomass, biogas, geothermal, small, and large hydro. The other 80% is selected in different ways to end up with 120% of the total annual load. In the added-wind cases, 60% is generated from scaling up the 2019 solar generation profile and the remaining 20% includes variable wind-solar (2019 solar) ratios as shown in the top right graph in Figure 7. It explains how we increase the wind ratio while decreasing the solar resulting in 20% of the total annual load. For example, added winter-dominant wind case (that has maximum added-wind) includes 40% historical generation +60% historical solar +20% winter-dominant wind. The historical solar share ranges between 60 and 80% when the added wind provides 0–20%. For the added-solar scenario, after the first 40%, the other 80% comes from the mix of 2019 solar and latitude-tilt or zero-tilt solar. We use variable solar-solar mix (2019 solar and latitude/zero tilt solar) by changing 5% at each step as shown in the bottom graph in Figure 7. For instance, the added latitude-tilt solar scenario (that has maximum latitude-tilt solar) includes 40% historical generation +80% latitude-tilt solar. The historical solar and added-solar make up this 80% by varying the fraction of each. The generation for the year was intentionally selected to be greater than the load (after accounting for losses) by 20%, as an example for demonstrating the methodology. This extra 20% of electricity may be curtailed or may be used for other applications and is seen during the calculation when the charging rate exceeds the charge rate limit or when storage is filled that is never emptied. As we are not certain about the total future generation, we do a sensitivity analysis using different amounts of total generation (i.e., 120%, 140%, 160% and 180% of annual load) in the supplementary section Figure S1 using a

generation mix from the baseline scenario. Not surprisingly, generating more electricity than is needed reduces the amount of storage that is required.

The offshore wind generation profiles sample sites in northern and southern California that are being considered for deployment.[26] The two onshore wind profiles show differences in annual generation shapes as described in.[27] Sites selected for onshore wind are based on existing generating plants. We take the average of four different sites in California for the two solar profiles. Table 1 lists the energy resources and modeling assumptions for this analysis. To summarize the specific locations of the sites selected for each generator, we add Table S1 in the supplementary section. Because these different types and locations of renewable electricity have substantially different generation profiles, the required amount and frequency of use of energy storage could significantly differ. We use RESOLVE candidate resources[28] and EIA 923[29] generation data for selecting sites. For each site, the NREL WIND toolkit[30] or PVWatts[31] is used to simulate the wind and solar output, respectively. All the wind calculations are done using 5-min interval data while we use hourly data for solar. A power curve of Aerodyn SCD 8.0/168 turbine and wind speed at 120-m height is used for the offshore scenarios. The onshore wind calculations use a GE 2.5–120 turbine power curve and 100-m wind speed.

Given the generation mixes described in Figure 7, we examine the amount and frequency of use of energy storage to balance the electricity demand and supply using the hierarchical approach described above. We assume energy storage bins with 80% round-trip efficiency are available to the grid. We also analyze the effect of different round trip efficiencies (i.e., 50% and 80%) on the minimum number of cycles and relative storage needs assuming a solar- or wind-dominated region. We show the impact of implementing a larger amount of wind on storage characteristics though it might not be feasible for the state of California to implement this amount of wind. However, our main purpose is to represent the application of our method and the results are not meant to be a specific solution to any region.

For the scope of this work, we did not consider any growth or modifiers in the demand profile, nor did we consider imports from other geographical regions. The method is intended to help evaluate any set of load and generation profiles, though the method in the presented form does not consider any transmission flows to and from adjacent regions. Also, it doesn't consider cost, capacity limits, and emission limits like capacity expansion modeling. We do not use any optimization in this hierarchical method; instead, we use different generation profiles as inputs, calculate generation and load balancing and determine the relative needs of storage along with their minimum annual frequency of cycling.

iScience, Volume *26*

Supplemental information

Hierarchical approach to evaluating

storage requirements

for renewable-energy-driven grids

Zabir Mahmud, Kenji Shiraishi, Mahmoud Y. Abido, Pedro Andrés Sánchez-Pérez, and Sarah R. Kurtz

Supplementary Material for "Hierarchical Approach to Evaluating Storage Requirements for Renewable-Energy-Driven Grids"

Zabir Mahmud[1], Kenji Shiraishi[2], Mahmoud Y. Abido[1, 3], P. A. Sánchez-Pérez[1], and Sarah Kurtz[1]

[1]University of California, Merced, 5200 N Lake Rd, Merced, CA 95343, United States
[2]University of California, Berkeley, 450 Sutardja Dai Hall, Berkeley, CA 94720, United States
[3]Cairo University, Giza, 1 Gamaa Street, Giza, Cairo 12613, Egypt

1 Energy Resources and Description of Modeled Sites

We explain how we consider sites for different scenarios. In Table S1, we mention the list of specific latitude and longitude with the region names modelled in the analysis.

Table S1: Description of the renewable electricity generators with their names, locations and modeling parameters. Related to STAR Method

	Modeled sites			
Energy resource	Name	Latitude	Longitude	Modeling assumptions
**Solar PV**				
Historical (2019)	CAISO data [1]	-	-	Module type: Standard,
Latitude tilt	Los Angeles	34.58	-118.16	Array type: 1-Axis tracking,
	Tehachapi	35.15	-118.44	Total system losses: 14.08%,
	Merced	37.29	-120.52	DC to AC ratio: 1.2,
				Data collected: 2012,
Zero tilt	Same locations as latitude tilt			Data type: hourly
**Onshore wind**				
Historical (2019)	CAISO data [1]	-	-	Turbine power curve: GE 2.5-120,
Summer dominant	Ocotillo	32.87	-115.55	
	San Gorgonio	33.88	-116.56	Hub height: 100m,
	Alta I	35.02	-118.25	Data collected: 2012,
	Shiloh I	38.12	-121.79	Data type: 5-min interval
Winter dominant	Golden Acorn Casino	32.70	-116.35	
	Tehachapi	35.14	-118.45	
	Carrizo	34.99	-120.15	
	Foundation Walmart	40.01	-122.13	
	Humboldt	40.76	-123.87	
**Offshore wind**				
Northern	Cape Mendocino	40.43	-124.41	Turbine power curve:
	Humboldt Bay	40.90	-124.37	Aerodyn SCD 8.0/168,
	Del Norte	41.63	-124.23	Hub height: 120m,
Southern	Diablo Canyon	35.13	-120.99	Data collected: 2019,
	Morro Bay	35.33	-121.01	Data type: 5-min interval

2 Sensitivity Analysis of Total Generation Amount

The generation is considered 120% of total annual load in the main paper. However, the total generation could be different in future. We analyze the impact of different generation level on energy storage statistics as shown in Figure S1. We see that the seasonal needs could be reduced by 4 times if the generation increases from 120% to 180% of total annual load though the effect on the required diurnal storage is relatively small.

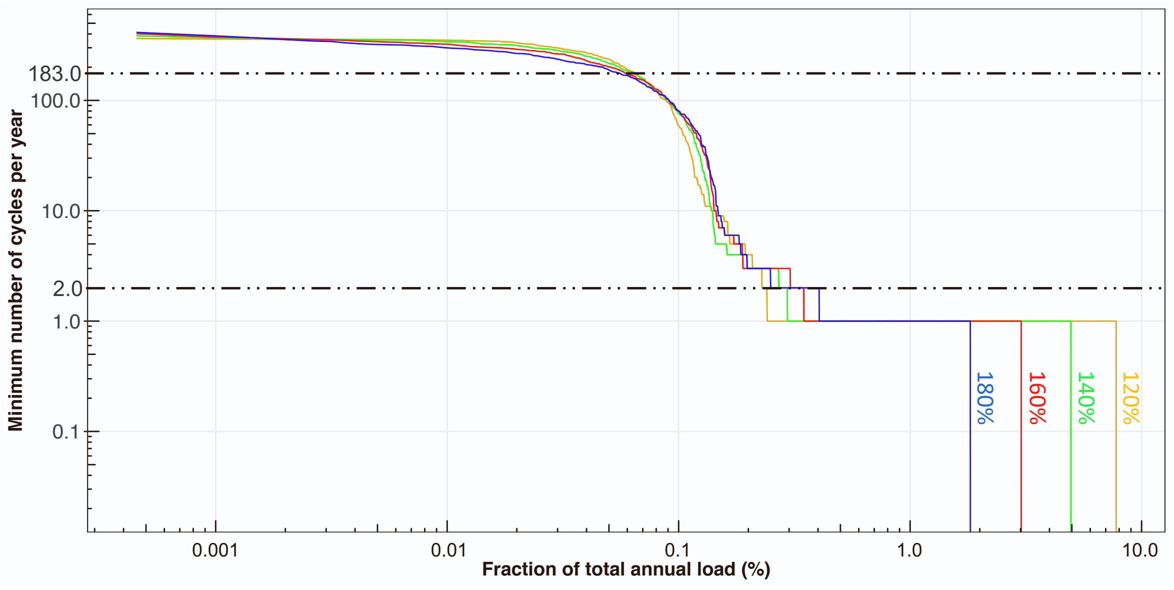

Figure S1: Sensitivity analysis of amount of total generation for baseline scenario energy mix. Related to Figure 1

3 General Equations

We calculate the eight generation profiles using Equations 1 and 2. Renewables include solar, wind, biomass, biogas, geothermal and small hydropower in Equation 2. Initially charge is calculated for each time step, t, by the difference between total generation and load as shown in Equation 3. Considering the charging and discharging efficiency, charges are recalculated using Equations 4 and 5. The state of charge of each storage bin is determined using the cumulative summation of charge after considering the dis/charging for 105,120 timesteps as shown in Equation 6.

$$\text{Total generation} = \text{Historical (2019) generation} + \text{Added generation} \tag{1}$$

$$\text{Historical (2019) generation} = \text{Renewables} + \text{Large hydro} \tag{2}$$

$$\text{Available Charge (t)} = \text{Total generation (t)} + \text{Load (t)} \quad t = \{1, 2, \ldots, 105120\} \tag{3}$$

$$\text{Charge (t)} = \text{Available charge (t)} \times \text{Charging efficiency} \tag{4}$$

$$\text{Charge (t)} = \text{Available charge (t)} \div \text{Discharging efficiency} \tag{5}$$

$$\text{State of charge (bin,t)} = \text{State of charge (bin,t-1)} + \text{Charge (t)} \tag{6}$$
